# Potential causal associations between adiposity and hydration status in children and adolescents: evidence from negative control and Mendelian randomization analyses

**DOI:** 10.3389/fnut.2026.1925090

**Published:** 2026-09-28

**Authors:** Yuwei Hou, Shuying Luo, Kaijuan Wang, Dazhong Wang, Yaodong Zhang

**Affiliations:** 1 Henan Key Laboratory of Children’s Genetics and Metabolic Diseases, Children’s Hospital Affiliated to Zhengzhou University, Henan Children's Hospital, Zhengzhou Children’s Hospital, Zhengzhou, Henan, China; 2 Department of Epidemiology and Health Statistics, College of Public Health, Zhengzhou University, Zhengzhou, Henan, China

**Keywords:** body mass index, children and adolescents, hydration status, Mendelian randomization, waist circumference

## Abstract

**Background:**

This study examined the potential causal association between body mass index (BMI), waist circumference (WC), and hydration status among children and adolescents.

**Methods:**

Participants aged 6–18 years were recruited from 10 cities in Henan Province, China, using a three-stage cluster random sampling strategy. BMI *Z*-scores and WC *Z*-scores were used as primary exposure variables. Hydration status was assessed using urine specific gravity (USG). Restricted cubic spline models were used to assess potential dose–response relationships between BMI *Z*-scores, WC *Z*-scores, and log-transformed USG. Logistic regression models were applied to estimate associations between adiposity indicators and hydration status. Parental BMI was used as negative control outcomes to assess potential residual confounding arising from shared familial factors. Bidirectional Mendelian randomization (MR) analyses were performed to investigate the potential causal effects.

**Results:**

A total of 11,935 children and adolescents (mean age 9.98 ± 2.17 years; 47.6% girls) were included. Both BMI *Z*-scores and WC *Z*-scores showed nonlinear associations with log-transformed USG. Higher BMI *Z*-scores (OR = 1.248, 95% CI: 1.011–1.541) and WC *Z*-scores (OR = 1.211, 95% CI: 1.109–1.322) were associated with increased risk of inadequate hydration. Negative control analyses showed no significant associations. MR analyses provided genetic evidence supporting potential causal effects of BMI (*β* = 0.073, 95% CI: 0.035–0.112) and WC (*β* = 0.097, 95% CI: 0.036–0.158) on USG, whereas reverse MR analyses, which were based on a limited number of available genetic instruments, found no evidence of reverse effects.

**Conclusion:**

Higher standardized BMI and WC were associated with an increased risk of inadequate hydration, with negative control and MR analyses providing supportive evidence for potential causality, while reverse causality remains uncertain.

## 1 Introduction

Maintaining an adequate hydration status is a fundamental basis for physiological homeostasis and normal bodily function (1). Water plays a central role in thermoregulation, nutrient transport, metabolic waste excretion, and cellular function maintenance (2). Previous studies have shown that disturbances in hydration status are not only associated with renal function and metabolic health (3), but are also linked to the development of chronic diseases such as diabetes (4), an increased risk of heart failure (5), and higher all-cause mortality (6). Although authoritative organizations such as the European Food Safety Authority (7) and the American Academy of Pediatrics (8) have provided clear recommendations for daily fluid intake, a substantial proportion of adolescents still fail to meet the recommended intake levels (9). A cross-sectional survey covering 13 countries reported a high prevalence of inadequate fluid intake across populations. The proportion of children and adolescents not meeting EFSA adequate intake recommendations varied widely by country, ranging from less than 20% in Uruguay and less than 40% in Brazil to over 80% in China and France, and over 90% in Belgium (10). Therefore, identifying key determinants of hydration status in adolescents is of great public health importance for promoting healthy development and developing targeted prevention and intervention strategies.

Among the various factors that may influence hydration status, obesity has increasingly attracted research attention. Obesity-related insulin resistance, chronic low-grade inflammation, and alterations in renal water and sodium regulation may impair urinary concentrating ability and overall fluid homeostasis (11, 12). Epidemiological studies further indicate that obesity-related indicators, such as body mass index (BMI), are significantly associated with hydration status (13, 14). Evidence from children and adolescents in Spain has shown that water intake standardized by body weight is positively associated with total body water, while being negatively associated with BMI and body fat mass (15), suggesting that individuals with obesity may be more prone to a relatively low hydration status. This issue is particularly important in adolescents. In recent years, the prevalence of overweight and obesity among children and adolescents has continued to rise globally. A systematic review and meta-analysis by Zhang et al. (16) reported that the overall prevalence of obesity among children and adolescents reached 8.5%, with prevalence rates of overweight, obesity, and combined overweight/obesity of 14.8 and 22.2%, respectively. Moreover, the prevalence of obesity nearly doubled between 2012 and 2023 compared with 2000–2011. In this context, if obesity indeed influences hydration status, weight management strategies may not only improve traditional cardiometabolic outcomes but may also serve as a potential approach to optimize hydration status in adolescents. Therefore, systematically evaluating the association between obesity and hydration status, and further clarifying its potential causal relationship, is of great importance for advancing adolescent health promotion strategies.

However, several limitations remain in studies examining the relationship between obesity and hydration status among children and adolescents. First, some studies have reported null or inconsistent associations between obesity and hydration status (14, 15, 17), which may be partly attributed to relatively small sample sizes and limited statistical power. Second, most existing studies are observational in nature and may not have adequately accounted for key confounding factors, such as family environment, dietary patterns, and lifestyle behaviors, which may bias the observed associations. Finally, the majority of findings is derived from cross-sectional designs. As a result, it remains unclear whether obesity leads to altered hydration status, whether hydration status influences obesity, or whether the observed association is driven by shared underlying factors. Robust evidence from causal inference approaches is still needed to clarify the direction of this relationship.

Therefore, based on a large, multi-regional dataset derived from an obesity screening program among Chinese children and adolescents, the present study aims to apply causal inference approaches to more precisely evaluate the association between obesity and hydration status and to assess its potential causal direction.

## 2 Methods

### 2.1 Study participants

Participants aged 6–18 years were recruited from the eastern, western, central, southern, and northern regions of Henan Province in China between June 2019 and September 2021. A uniform protocol was implemented across all participating schools. To ensure sample representativeness, a three-stage cluster random sampling strategy was employed. First, 10 cities were selected from Henan Province using probability-proportional-to-size sampling. Second, six schools were randomly chosen from each city. Third, six classes were randomly selected from each school, and all students in these classes were enrolled. Written informed consent was obtained from both the children/adolescents and their parents or legal guardians before the start of the survey. Participants were informed that they could refuse or withdraw at any time without consequence. This study was approved by the Ethics Committee of Henan Children’ s Hospital (Approval No. 2021-H-K30).

A standardized questionnaire was used to collect data on children and adolescent overweight/obesity, encompassing socio-demographic characteristics, daily sleep duration, physical activity, screen time, and eating habits. The questionnaires were distributed in class for students to take home to their parents or caregivers. Detailed instructions accompanied the questionnaire to guide parents in its completion and ensure data accuracy. Trained investigators subsequently collected the completed forms from each classroom. The children and adolescents underwent physical examinations and provided urine and blood samples. All completed questionnaires were subject to quality checks. A total of 19,312 children and adolescents aged 6–18 years were initially included in this study. After excluding participants with missing or implausible values for height, weight, and waist circumference (WC), 12,481 participants remained. After further excluding those with missing urine samples, 11,936 participants were retained. Finally, after excluding participants with missing covariates, a total of 11,935 participants were included in the final analysis. The participant selection process is shown in Supplementary Figure S1.

### 2.2 Anthropometric measurements and obesity definitions

Standing height (cm), body weight (kg), and WC (cm) were measured on site for each participant. BMI was calculated as weight divided by height squared (kg/m^2^). Because BMI and WC are strongly influenced by age and sex, their raw values are not directly comparable across children of different ages and sexes. Therefore, age- and sex-specific standardization was applied to BMI and WC in the statistical analyses. BMI *Z*-scores were calculated according to the WHO 2007 growth reference for children and adolescents using the LMS (Lambda–Mu–Sigma) method implemented in the R package *anthroplus* (18). As no widely accepted international reference for WC is available, WC *Z*-scores were calculated using an internal standardization approach by sex and age in 1-year groups, with the mean and standard deviation calculated within each subgroup. General obesity and abdominal obesity were defined according to age- and sex-specific BMI and WC cut-off values, respectively, based on the 2018 guidelines issued by the National Health Commission of China (WS/T 586—2018; WS/T 611—2018) (19, 20). In the statistical analyses, continuous BMI *Z*-scores and WC *Z*-scores were used as primary exposure variables, whereas binary indicators of general and abdominal obesity were included in sensitivity analyses.

### 2.3 Assessment of hydration status

Urine specific gravity (USG) is commonly used as a surrogate indicator of hydration status, as it indirectly reflects the concentration of solutes in urine. Due to its simplicity, rapid measurement, and low cost, USG has been applied in clinical practice and epidemiological studies. In this study, spot urine samples (midstream of the first morning void) were collected on-site by trained investigators following standardized procedures. Participants were instructed to avoid vigorous physical activity and excessive fluid intake prior to sample collection and to provide midstream urine samples in the morning as instructed. All urine samples were collected in pre-labeled sterile containers, initially recorded on-site, temporarily stored at 2–8 °C, and promptly transported to the certified laboratory. Upon arrival at the laboratory, samples were immediately aliquoted and stored at −20 °C until batch analysis to minimize the effects of repeated freeze–thaw cycles and prolonged exposure to room temperature. Urine specific gravity (USG) was measured using an automated urine analyzer (MUS-9600, DIRUI, Changchun, China). Based on prior evidence and the observed distribution of USG among children and adolescents in this study (21, 22), urine specific gravity was classified into two hydration categories: adequate hydration (USG 1.000–1.020) and inadequate hydration (USG > 1.020).

### 2.4 Covariates

For the present analysis, a range of sociodemographic and lifestyle factors were considered as covariates. Age (years) was treated as a continuous variable, and sex was classified as boy or girl. Parental socioeconomic status was captured by paternal and maternal educational level (primary or junior high school, senior high school, college or above) and household income level [≤50,000 Chinese yuan (CNY), 50,000–150,000 CNY, > 150,000 CNY]. Lifestyle behaviors included breakfast frequency (rarely or never, 1–3 days/week, 4–5 days/week, every day), screen time (<0.5, 0.5–2, ≥2 h/day), sleep duration (<8, 8–9, 9–10, ≥10 h/day), exercise frequency (almost none, 1–3, 4–6 times/week, every day), and total exercise duration per week (<90, 90–119, 120–149, ≥150 min). Unhealthy dietary behaviors were additionally assessed using questionnaire items on late-night eating and snack consumption. Participants were asked whether they habitually consumed food at night in addition to regular meals and whether they frequently consumed snacks.

### 2.5 Statistical analysis

#### 2.5.1 Descriptive statistical analysis and regression analyses

For continuous variables, the data were summarized as means and standard deviations if they were normally distributed, and as medians with interquartile ranges if they were not. Between-group differences were assessed using Student’s *t* test or the Wilcoxon rank-sum test. Categorical variables were presented as frequencies and percentages, and group comparisons were performed using the chi-square test. Ordered categorical variables were compared using the Kruskal–Wallis rank-sum test, with the test statistic reported as the *H* statistic.

Restricted cubic splines (RCS) with four knots were first used to examine the dose–response relationships of BMI *Z*-scores and WC *Z*-scores with log-transformed USG. Segmented regression models and Davies test were applied to assess the presence of potential threshold effects and to identify inflection points in the dose–response associations. Logistic regression models were then fitted to assess the association between BMI *Z*-scores, WC *Z*-scores and hydration status among children and adolescent hydration status, and results were expressed as odds ratios (ORs) with 95% confidence intervals (CIs). A series of four models were constructed. Model 1 was a crude model without covariate adjustment. Model 2 additionally adjusted for age, gender, paternal and maternal educational level, household income level, breakfast frequency, screen time, sleep duration, weekly exercise frequency, and total exercise duration per week. Model 3 further adjusted for late-night eating habit and snack consumption habit. Model 4 was constructed using stabilized inverse probability weighting (IPW) to balance covariates across the distribution of BMI *Z*-scores and WC *Z*-scores. To account for the multistage cluster sampling design, all regression models were fitted with robust standard errors clustered at the school level. Finally, subgroup analyses were conducted to explore potential effect modifiers.

#### 2.5.2 Negative control analysis for residual confounding

Negative control exposure (NCE) and negative control outcome (NCO) methods were proposed by Lipsitch et al. (23) in 2010 to detect bias arising from unmeasured confounding through the use of auxiliary variables that are not causally related to the study outcome or exposure. In the present study, parental BMI was initially considered for negative control analysis. However, parental BMI may influence child BMI through genetic background pathways and therefore does not satisfy the assumption that the NCE should not affect the study outcome. Accordingly, an NCO framework was adopted.

Specifically, child hydration status was temporarily treated as the exposure, child BMI as the outcome, and parental BMI as the negative control outcome. Parental BMI and child BMI share important familial sources of confounding, including genetic background, dietary patterns, socioeconomic conditions, and lifestyle factors. In contrast, child hydration status is not expected to causally influence parental BMI. Under this framework, an association between child hydration status and parental BMI after adjustment for measured covariates would provide evidence suggestive of residual confounding shared between parents and offspring. It is worth noting that parental BMI was not assumed to fully capture all unmeasured confounding underlying the association between child adiposity and hydration status. Therefore, the NCO analysis was used solely as a qualitative bias-detection approach to assess whether residual familial confounding could plausibly contribute to the observed associations. A detailed derivation of the NCO model is provided in Supplementary Method S1.

#### 2.5.3 Bidirectional Mendelian randomization analysis

To further clarify the causal direction between adiposity and hydration status, bidirectional Mendelian randomization (MR) analyses were performed. To minimize potential bias arising from sample overlap, summary-level genome-wide association study (GWAS) data from individuals of European ancestry were used. BMI GWAS data were obtained from Loh et al. (24) (*N* = 532,396), and WC GWAS data were derived from Mbatchou et al. (25) (*N* = 407,661). In the forward MR analyses, single nucleotide polymorphisms (SNPs) associated with BMI and WC at genome-wide significance (*p* < 5 × 10^−8^) were selected as instrumental variables. In reverse MR analyses, owing to the limited availability of genetic instruments for USG, GWAS data reported by Dennis et al. (26) were used (*N* = 40,547). In this original GWAS, repeated USG measurements were summarized as the median value for each individual, and the median USG values were subsequently subjected to inverse normal quantile transformation (INT) to account for skewness and non-normality. The resulting INT-transformed USG values were used as the phenotype for GWAS. Given the limited number of genome-wide significant instruments, the SNP selection threshold was relaxed to *p* < 1 × 10^−5^. All SNPs were clumped based on linkage disequilibrium (LD) criteria (LD *R^2^* < 0.001, LD distance > 10,000 kb) to ensure independence. Instrument strength was evaluated using the *F* statistic, with *F* > 10 indicating sufficient strength. The random-effects inverse-variance weighted (IVW) method was applied as the primary analysis to estimate the overall causal effects of genetically predicted exposures on outcomes (27). Cochran’s *Q* statistic and the *I^2^* index were used to assess heterogeneity among instruments, while the MR-Egger intercept test was performed to evaluate directional horizontal pleiotropy. To assess robustness, several complementary MR approaches were conducted, including MR-Egger regression (28), weighted median estimation (29), contamination mixture method (ConMix) (30), MR-robust adjusted profile score (MR-RAPS) (31), MR-pleiotropy residual sum and outlier (MR-PRESSO) (32), and constrained maximum likelihood and model averaging Bayesian information criterion (cML-MA-BIC) (33). Detailed descriptions of MR assumptions and MR approaches are provided in Supplementary Method S2.

#### 2.5.4 Sensitivity analysis

To assess the robustness of the findings, several sensitivity analyses were conducted. First, considering the potential influence of the selected cutoff of USG > 1.020 for defining inadequate hydration, USG was analyzed as a continuous outcome after natural log transformation (log-USG), and the associations of BMI *Z*-scores and WC *Z*-scores with log-USG were re-estimated. Second, to reduce the influence of extreme observations, participants with values below the 2.5th percentile or above the 97.5th percentile for any of USG, BMI *Z*-scores, or WC *Z*-scores were excluded, and all regression models were re-estimated in the restricted dataset. Third, for the reverse-direction MR analyses, sensitivity analyses were additionally performed using more stringent *p*-value thresholds for SNP selection. Fourth, leave-one-out analyses were conducted for all MR analyses by sequentially excluding each SNP from the instrumental variable set to assess whether any single SNP disproportionately influenced the overall effect. All statistical tests were two-sided and a *p* value of less than 0.05 was considered statistically significant. All analyses were performed using R software (version 4.5.1).

## 3 Results

### 3.1 Baseline characteristics of study participants

A total of 11,935 children and adolescents were included in this study, with a mean age of 9.98 ± 2.17 years, and 47.6% were girls. The proportions of fathers and mothers with an education level of college or above were 47.2 and 44.9%, respectively, and the vast majority of households (87.5%) had an annual income below 150,000 CNY. Overall, 89.4% of participants ate breakfast every day, 92.9% had a daily screen time of less than 0.5 h, 7.5% slept fewer than 8 h per night, 13.2% reported a late-night eating habit and 78.6% reported a snack consumption habit. Scatter plots showed clear positive correlations between BMI and WC (*r* = 0.691, *p* < 0.001) and between their corresponding *Z*-scores (*r* = 0.282, *p* < 0.001, Supplementary Figure S2). Based on hydration status criteria, 2,582 children and adolescents were classified as having inadequate hydration, accounting for 21.63% of the study population. Between-group comparisons revealed significant differences in BMI, WC and their *Z*-scores among children and adolescents, as well as in gender, paternal and maternal educational level, breakfast frequency, screen time, exercise frequency, and total exercise duration (Table 1).

**Table 1:** Baseline characteristics of study participants grouped by hydration status.

Variables	Overall (*n* = 11,935)	Adequate hydration (*n* = 9,353)	Inadequate hydration (*n* = 2,582)	*t/H/χ^2^*	*p*
Continuous variable, mean ± SD/median (IQR)					
Age (years)	9.98 ± 2.17	9.98 ± 2.18	9.98 ± 2.11	0.123	0.094
Adolescent BMI (kg/m^2^)	20.90 ± 1.48	20.86 ± 1.45	21.06 ± 1.57	−5.903	<0.001
Adolescent BMI *Z*-score	1.69 ± 0.63	1.68 ± 0.64	1.73 ± 0.62	−4.275	<0.001
Adolescent WC (cm)	59.07 ± 10.14	58.63 ± 9.94	60.64 ± 10.69	−8.566	<0.001
Adolescent WC *Z*-score	0 ± 1.00	−0.04 ± 0.97	0.15 ± 1.08	−8.373	<0.001
Paternal BMI (kg/m^2^)	25.05 ± 2.89	25.05 ± 2.88	25.06 ± 2.94	−0.216	0.828
Maternal BMI (kg/m^2^)	22.80 ± 2.84	22.78 ± 2.83	22.88 ± 2.87	−1.620	0.105
Categorical variable, *n* ± %					
Gender				7.224	0.007
Boy	6,250 (52.4)	4,837 (51.7)	1,413 (54.7)		
Girl	5,685 (47.6)	4,516 (48.3)	1,169 (45.3)		
Paternal educational level				12.687	<0.001
Primary or junior high	3,161 (26.5)	2,433 (26.0)	728 (28.2)		
Senior high school	3,140 (26.3)	2,420 (25.9)	720 (27.9)		
College or above	5,634 (47.2)	4,500 (48.1)	1,134 (43.9)		
Maternal educational level				19.414	<0.001
Primary or junior high	3,421 (28.7)	2,608 (27.9)	813 (31.5)		
Senior high school	3,161 (26.5)	2,457 (26.3)	704 (27.3)		
College or above	5,353 (44.9)	4,288 (45.8)	1,065 (41.2)		
Household income level				0.489	0.484
≤50,000 CNY	4,190 (35.1)	3,258 (34.8)	932 (36.1)		
50,000–150,000 CNY	6,253 (52.4)	4,935 (52.8)	1,318 (51.0)		
>150,000 CNY	1,492 (12.5)	1,160 (12.4)	332 (12.9)		
Breakfast frequency (times/week)				14.195	<0.001
Rarely or never	227 (1.9)	173 (1.8)	54 (2.1)		
1–3 days/week	349 (2.9)	258 (2.8)	91 (3.5)		
4–5 days/week	687 (5.8)	506 (5.4)	181 (7.0)		
Every day	10,672 (89.4)	8,416 (90.0)	2,256 (87.4)		
Screen time (hours/day)				4.019	0.044
<0.5 h	11,083 (92.9)	8,708 (93.1)	2,375 (92.0)		
0.5–2 h	563 (4.7)	434 (4.6)	129 (5.0)		
≥2 h	289 (2.4)	211 (2.3)	78 (3.0)		
Sleep duration (hours/day)				1.327	0.249
<8 h	899 (7.5)	701 (7.5)	198 (7.7)		
8–9 h	5,539 (46.4)	4,317 (46.2)	1,222 (47.3)		
9–10 h	4,418 (37.0)	3,483 (37.2)	935 (36.2)		
≥10 h	1,079 (9.0)	852 (9.1)	227 (8.8)		
Exercise frequency (sessions/week)				4.258	0.039
Almost none	1725 (14.5)	1,314 (14.0)	411 (15.9)		
1–3 times/week	6,045 (50.6)	4,751 (50.8)	1,294 (50.1)		
4–6 times/week	1,674 (14.0)	1,309 (14.0)	365 (14.1)		
Every day	2,491 (20.9)	1979 (21.2)	512 (19.8)		
Total exercise duration				17.126	<0.001
<90 min	4,680 (39.2)	3,589 (38.4)	1,091 (42.3)		
90–119 min	3,290 (27.6)	2,593 (27.7)	697 (27.0)		
120–149 min	1,440 (12.1)	1,118 (12.0)	322 (12.5)		
≥150 min	2,525 (21.2)	2053 (22.0)	472 (18.3)		
Late-night eating habit				<0.001	0.999
No	10,358 (86.8)	8,117 (86.8)	2,241 (86.8)		
Yes	1,577 (13.2)	1,236 (13.2)	341 (13.2)		
Snack consumption habit				3.173	0.074
No	2,559 (21.4)	1972 (21.1)	587 (22.7)		
Yes	9,376 (78.6)	7,381 (78.9)	1995 (77.3)		

### 3.2 Association between BMI *Z*-scores, WC *Z*-scores and hydration status

Figure 1 shows that BMI *Z*-scores and WC *Z*-scores were nonlinearly associated with log-transformed USG (both *P* for nonlinearity <0.001). However, Davies’ tests did not provide evidence of statistically significant changes in slope for either BMI *Z*-scores or WC *Z*-scores. The estimated optimal breakpoints were 2.863 for BMI *Z*-scores (*p* = 0.106) and −1.716 for WC *Z*-scores (*p* = 0.126). Table 2 presents the logistic regression results for the associations of BMI *Z*-scores and WC *Z*-scores with inadequate hydration among children and adolescents. In the IPW models, each 1-unit increase in BMI *Z*-score and WC *Z*-score was associated with a 24.8 and 21.1% higher risk of inadequate hydration, respectively (BMI *Z*-scores: OR = 1.248, 95% CI: 1.011–1.541; WC *Z*-scores: OR = 1.211, 95% CI: 1.109–1.322).

**Figure 1:**
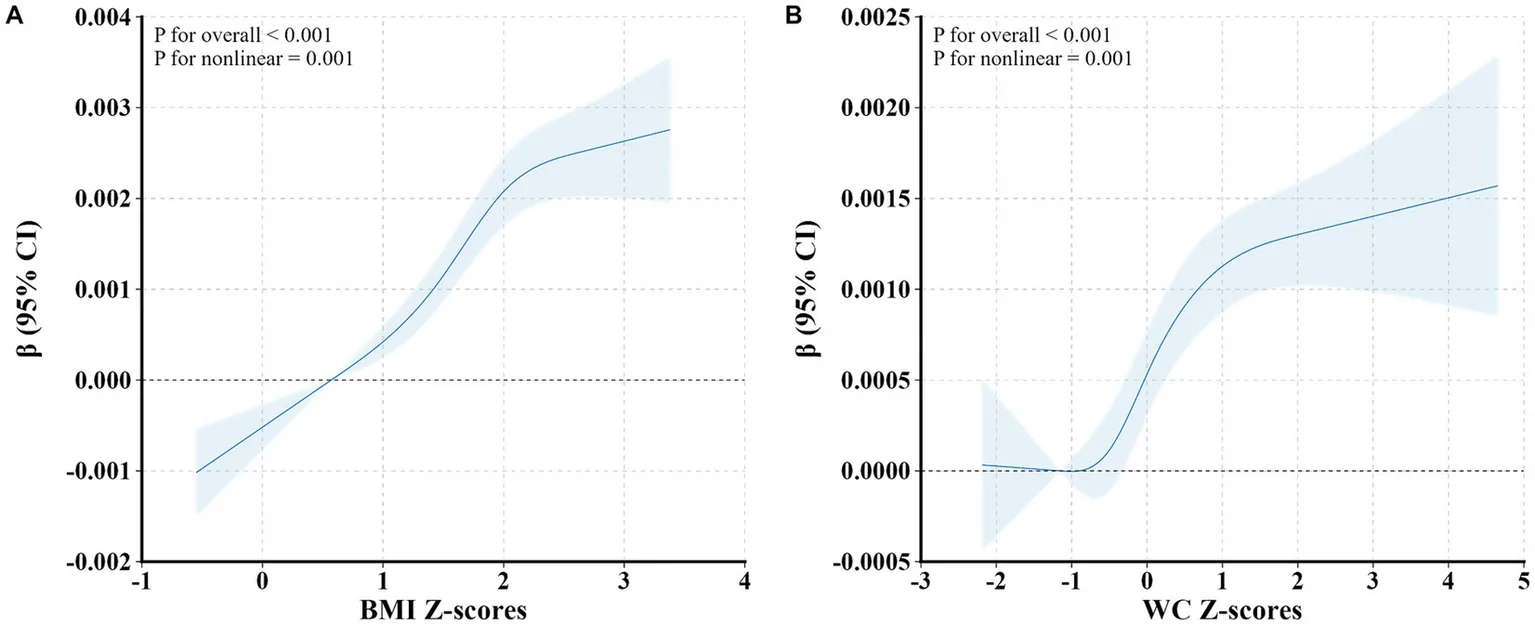
Restricted cubic spline dose–response relationships of BMI *Z*-scores **(A)** and WC *Z*-scores **(B)** with log-transformed USG in children and adolescents.

**Table 2:** Associations between BMI *Z*-scores, WC *Z*-scores and hydration status among children and adolescents.

Model	BMI *Z*-scores			WC *Z*-scores		
	OR	95% CI	*p*	OR	95% CI	*p*
Model 1	1.162	0.975–1.384	0.093	1.205	1.099–1.322	<0.001
Model 2	1.482	1.047–2.099	0.027	1.199	1.105–1.302	<0.001
Model 3	1.471	1.041–2.079	0.029	1.197	1.104–1.298	<0.001
Model 4	1.248	1.011–1.541	0.040	1.211	1.109–1.322	<0.001

OR, odds ratio; CI, confidence interval. Model 1 was unadjusted. Model 2 was fitted with adjustment for covariates including sex, age, parental education, income, breakfast frequency, screen time, sleep duration, weekly exercise frequency, and total exercise duration. Model 3 additionally adjusted for late-night eating habits and snack consumption habits beyond Model 2 covariates. Model 4 was based on stabilized IPW models. All models were estimated using robust standard errors clustered at the school level to account for the multistage sampling design.

### 3.3 Negative control analyses

Supplementary Figure S3 showed that BMI in children and adolescents was weakly but significantly correlated with paternal BMI, maternal BMI, and the mean of parental BMI. As shown in Supplementary Table S1, no significant associations were observed between children and adolescent’s hydration status and negative control outcomes. This analysis did not provide evidence that the observed associations between BMI *Z*-scores and hydration status among children and adolescents were driven by the shared familial factors captured by parental BMI.

### 3.4 Mendelian randomization analyses

In the forward MR analyses, 297 and 166 independent SNPs were used as instrumental variables for BMI and WC, respectively. The mean and minimal *F*-statistics were 71.817 and 27.639 for BMI, and 63.288 and 29.786 for WC, indicating strong instrument strength. Detailed characteristics of the genetic instruments are presented in Supplementary Tables S2, S3. Table 3 presents the MR results for the associations of BMI and WC with USG. For BMI, the results of IVW model suggested a significant positive association with USG (*β* = 0.073, 95% CI: 0.035–0.112). This finding was consistent across multiple sensitivity analyses, including MR-Egger, weighted median, ConMix, MR-PRESSO, MR-RAPS, and cML-MA-BIC, supporting the robustness of the association. No evidence of directional pleiotropy was detected (MR-Egger intercept = −0.001, *p* = 0.303). Similarly, WC was also positively associated with USG in the IVW model (*β* = 0.097, 95% *CI*: 0.036–0.158, *p* = 0.002). Consistent results were observed in multiple sensitivity analyses. The MR-Egger intercept test indicated no evidence of horizontal pleiotropy (intercept = −0.002, *p* = 0.110).

**Table 3:** Mendelian randomization estimates for the causal effects of BMI and WC on USG.

Exposures	Methods	*β* (95% *CI*)	*p*	*Q*	*I* ^2^	Intercept
BMI	IVW	0.073 (0.035–0.112)	<0.001	282.323	0%	−0.001 (*p* = 0.303)
	MR-Egger	0.119 (0.023–0.216)	0.015			
	Weighted median	0.083 (0.018–0.149)	0.012			
	ConMix	0.085 (0.025–0.145)	0.009			
	MR-PRESSO	0.075 (0.038–0.113)	<0.001			
	MR-RAPS	0.073 (0.034–0.113)	<0.001			
	cML-MA-BIC	0.069 (0.018–0.119)	0.008			
WC	IVW	0.097 (0.036–0.158)	0.002	135.417	0%	−0.002 (*p* = 0.110)
	MR-Egger	0.210 (0.060–0.360)	0.002			
	Weighted median	0.157 (0.058–0.255)	0.007			
	ConMix	0.211 (0.061–0.291)	0.002			
	MR-PRESSO	0.104 (0.051–0.157)	<0.001			
	MR-RAPS	0.101 (0.043–0.159)	<0.001			
	cML-MA-BIC	0.093 (0.022–0.164)	0.010			

CI, confidence interval; cML-MA-BIC, constrained maximum likelihood and model averaging Bayesian information criterion; ConMix, contamination mixture; IVW, inverse-variance weighted; MR-PRESSO, MR pleiotropy residual sum and outlier; MR-RAPS, MR robust adjusted profile score; USG, urine specific gravity.

In the reverse MR analyses, 16 independent SNPs were used as instrumental variables for USG. The mean and minimal *F*-statistics were 21.019 and 19.512. Detailed characteristics of the genetic instruments are provided in Supplementary Table S4. As shown in Supplementary Table S5, the IVW model showed no evidence of an association of genetically predicted USG with BMI (*β* = −0.038, 95% CI: −0.101 to 0.026) or WC (*β* = −0.004, 95% CI: −0.057 to 0.049). Consistent null findings were observed across all sensitivity analyses. No evidence of directional pleiotropy was detected based on the MR-Egger intercept tests. Because the units and standardization of BMI, WC, and USG in the GWAS datasets were not fully clear, the MR estimates should be interpreted as providing directional evidence rather than clinical effect-size estimates.

### 3.5 Subgroup analysis

Supplementary Figures S4, S5 presented the subgroup analyses of the associations of BMI *Z*-scores and WC *Z*-scores with hydration status in children and adolescents, respectively. While most associations were consistent, non-significant results were observed in some levels of breakfast frequency, screen time, and sleep duration, suggesting possible effect modification by these lifestyle factors in the associations between BMI/WC *Z*-scores and hydration status.

### 3.6 Sensitivity analysis

First, the associations of BMI *Z*-scores and WC *Z*-scores with continuous log-USG were consistent with those observed in the main analyses (Supplementary Table S6). Second, after excluding participants with extreme values, the associations between BMI *Z*-scores, WC *Z*-scores, and hydration status remained largely unchanged (Supplementary Table S7). Third, consistent findings were also observed in the corresponding NCO analyses (Supplementary Table S8). Fourth, in the reverse-direction MR sensitivity analyses, no SNPs were available when applying a selection threshold of *p* < 1 × 10^−6^. The threshold was subsequently relaxed to *p* < 5 × 10^−6^, which yielded 16 SNPs, and 8 independent SNPs remained after LD clumping. The MR estimates based on this instrument set were consistent with the main analysis (Supplementary Table S9). Fifth, all IVs had *F*-statistics greater than 20 (Supplementary Table S10). As illustrated in Supplementary Figures S6, S7, sequential exclusion of individual SNPs did not materially alter the overall IVW estimates for the associations of BMI and WC with urine specific gravity. In the reverse MR analyses, leave-one-out analyses similarly showed stable effect estimates following the removal of each SNP (Supplementary Figures S8–S11).

## 4 Discussion

In this study, using a large, multi-regional cross-sectional survey dataset, we systematically evaluated the associations between BMI *Z*-scores, WC *Z*-scores and hydration status among children and adolescents. The results showed that BMI *Z*-scores and WC *Z*-scores were significantly associated with higher risks of inadequate hydration assessed by USG. The negative control analysis, performed as a supplementary bias-detection analysis, did not provide evidence that our main findings were driven by the shared familial factors captured by parental BMI. Furthermore, MR analyses provided additional genetic evidence supporting potential causal effects of BMI and WC on hydration status, while the reverse MR analyses provided no evidence of reverse associations.

The core findings of this study are broadly consistent with prior evidence suggesting an association between adiposity and hydration status. For example, Carretero-Gómez et al. (34) assessed hydration status in an outpatient population using plasma osmolality, USG, and urine osmolality, and found that higher body weight status was consistently associated with increased plasma osmolality, urine osmolality, and USG, with plasma osmolality showing the strongest and most consistent relationship across BMI categories. Although their sample size and age structure differed from those of our study, the use of multiple hydration metrics may have reduced outcome misclassification. Similarly, Celik et al. (35) reported in an adolescent case–control study that adolescents with obesity were generally less hydrated than their normal-weight counterparts. Laja García et al. (15) evaluated water intake among Spanish adolescents aged 12–18 years using a validated hydration status questionnaire and estimated body composition via bioelectrical impedance analysis, showing moderate inverse associations of BMI and fat mass with water intake. Clayton et al. (17) used 24-h urine-collected USG and assessed body composition using DEXA in healthy children and adolescents, finding total water intake was positively associated with lean mass. Taken together, these studies and our findings collectively suggest that obesity in children and adolescents may be related to inadequate hydration.

Compared with the studies cited above, our study attempted to minimize potential biases through multiple design and analytic strategies. First, the use of a large, multi-regional dataset reduced random error, while also partially mitigating selection bias and limited generalizability commonly observed in single-center or highly selected populations, thereby enhancing the representativeness of our findings. In the statistical analyses, we applied stabilized IPW to balance sociodemographic and lifestyle characteristics across the distribution of BMI *Z*-scores and WC *Z*-scores. This approach aimed to approximate the exchangeability assumption by constructing weighted pseudo-populations with comparable covariate distributions, thereby providing marginal effect estimates complementary to conventional covariate-adjusted models. Second, we conducted negative control analyses using parental BMI and derived obesity indicators to probe potential unmeasured confounding arising from shared familial factors. Similar approaches have been widely used in prior research. For instance, Santos Ferreira et al. (36) used paternal BMI as a negative control and suggested that associations between maternal BMI and offspring metabolic profiles may largely reflect shared genetic or familial lifestyle confounding rather than intrauterine mechanisms. Lim et al. (37) used paternal pregnancy BMI as negative control exposures, providing support for a possible intrauterine effect of high maternal pre-pregnancy BMI on offspring outcomes. Likewise, in our study, children’s and adolescents’ hydration status is unlikely to have a direct physiological effect on parental BMI or obesity; however, shared family environments and genetic background may introduce family-level confounding, including genetic susceptibility, dietary behaviors, and socioeconomic context. Therefore, by temporarily reversing the roles of exposure and outcome, we constructed a negative control outcome framework that better satisfies the required assumptions. Finally, and importantly, we conducted bidirectional Mendelian randomization analyses to evaluate potential causality. By leveraging genetic variants as instrumental variables that are largely independent of environmental and behavioral confounding, MR provides quasi-experimental evidence complementary to observational analyses. Notably, the reverse MR analyses were limited by the small number of suitable independent SNPs available as genetic instruments, which was insufficient to provide strong evidence against reverse causality.

Based on current evidence, the association between obesity and insufficient hydration may be partly explained by chronically elevated arginine vasopressin (AVP) levels in obese individuals. AVP is a key hypothalamic peptide hormone that plays a central role in body fluid homeostasis, promoting renal water reabsorption to maintain fluid balance and inducing vasoconstriction to regulate blood pressure in response to increased plasma osmolality or reduced blood volume (38). AVP and copeptin are co-secreted in a 1: 1 molar ratio, making copeptin a stable and reliable surrogate marker of AVP secretion (12). Consistently, case–control studies in both children (39) and adults (40) have demonstrated significantly higher copeptin levels in individuals with obesity compared with lean controls. The elevation of AVP in obesity may be driven by multiple interacting mechanisms. First, obesity is frequently accompanied by insulin resistance, and elevated insulin levels can directly stimulate hypothalamic paraventricular and supraoptic nuclei, thereby promoting AVP secretion. In turn, AVP enhances hepatic glucose output, exacerbating hyperglycemia and insulin resistance, forming a potential feed-forward loop that sustains elevated AVP levels in obesity (41). Supporting the clinical relevance of hydration-related metabolic dysregulation, a large prospective cohort study by Dong et al. involving 71,526 Chinese adults demonstrated that, compared with the reference group, individuals classified as impending dehydration, dehydration, and severe dehydration exhibited 16, 26, and 33% higher risks of incident type 2 diabetes, respectively (22). Second, obesity is characterized by marked activation of the sympathetic nervous system (SNS) and the renin–angiotensin–aldosterone system (RAAS), which represent key mechanisms underlying obesity-related hypertension (42). SNS activation can directly stimulate AVP release, while angiotensin II, the major effector peptide of the RAAS, acts as a potent AVP secretagogue that promotes both hypothalamic synthesis and secretion of AVP (43). Finally, chronic low-grade inflammation associated with obesity may contribute to AVP system upregulation through activation of the hypothalamic–pituitary–adrenal (HPA) axis. In inflammatory states, multiple cytokines stimulate corticotropin-releasing hormone release, thereby activating the HPA axis and increasing cortisol secretion. Meanwhile, AVP, as a stress-related neuropeptide, acts synergistically with corticotropin-releasing hormone at the anterior pituitary to enhance adrenocorticotropic hormone release, thereby amplifying adrenal cortisol production (44). This concept of AVP-mediated co-activation of the HPA axis has been proposed as a potential mechanism in the pathophysiology of metabolic syndrome (45). Supporting this, Schinke et al. demonstrated in a dexamethasone/corticotropin-releasing hormone challenge test that obese individuals exhibit elevated copeptin levels, which are associated with enhanced HPA axis responsiveness characterized by increased adrenocorticotropic hormone and cortisol secretion (46). In addition, similar finding has been reported in pediatric populations (40), where obese children show significantly higher 24-h urinary free cortisol levels, which suggests that their HPA axis may be persistently activated in the early stages of obesity.

An additional finding of this study was that both BMI *Z*-scores and WC *Z*-scores, as two distinct adiposity indicators, were significantly and non-linearly associated with log-transformed USG, and exhibited highly similar dose–response patterns, which has not been reported in previous studies. Although the Davies’ tests did not identify statistically significant inflection points, the dose–response curves nevertheless suggested a pattern of rapid increase in USG at lower levels of adiposity, followed by a more gradual increase at higher levels of BMI *Z*-scores and WC *Z*-scores. The observed nonlinear association may be attributable to physiological limits in renal tubular transport capacity. As described by Esteva-Font et al. (47), the maximal urinary concentrating ability of the kidney is constrained by the expression and functional capacity of water channels and sodium transporters. Although obesity-related increases AVP initially enhances solute reabsorption and increases urinary concentration, this effect tends to stabilize once renal tubular transport reaches its maximal functional capacity. However, this proposed mechanism remains speculative, as AVP, osmolality, and renal tubular function were not directly assessed in the present study. Further studies incorporating direct measurements of these physiological markers are needed to clarify the mechanisms underlying the observed nonlinear association.

Despite the strengths of this study, several limitations should be acknowledged. First, because this was a cross-sectional study with concurrent measurement of exposure and outcomes, temporal ordering could not be established. Prospective studies are needed to confirm directionality. Second, hydration status was assessed using a single biomarker, which may be affected by short-term fluid intake and timing of sampling, leading to potential measurement error. Future work should incorporate additional markers, such as plasma osmolality, or repeated measurements. Third, because no LMS reference standard is currently available for age- and sex-specific WC in children and adolescents, we used an internal standardization approach based on the study population. Therefore, WC-based measures of abdominal obesity were population-specific. Fourth, the available genetic instruments for USG were primarily derived from European ancestry populations rather than Chinese populations, and the GWAS populations did not specifically comprise children or adolescents. Moreover, the limited number of genetic instruments available for reverse MR reduced statistical power, making it difficult to sufficiently exclude reverse causation. Therefore, the MR findings should be interpreted with caution. Fifth, we did not collect data on daily fluid intake. Daily fluid intake is a direct determinant of USG and may also be associated with BMI, dietary intake, and lifestyle behaviors. Therefore, the lack of information on fluid intake may have resulted in residual confounding. Future studies should incorporate detailed dietary records or 24-h fluid intake recalls to better isolate the specific impact of adiposity on hydration from overall fluid consumption.

## 5 Conclusion

In this large multi-regional study of children and adolescents, higher BMI *Z*-scores and WC *Z*-scores were associated with an increased risk of inadequate hydration assessed by USG. Evidence from negative control and Mendelian randomization analyses provided complementary evidence consistent with a potential effect, whereas the possibility of reverse causation remains unresolved and requires more robust evidence. Future longitudinal studies incorporating repeated assessments and multiple hydration biomarkers are warranted to validate these findings and elucidate the underlying mechanisms.

## Data Availability

The datasets presented in this article are not readily available because this data pertains to the privacy of the patients and is not suitable for public disclosure or for being provided without reservation to others. Requests to access the datasets should be directed to Yaodong Zhang, ydzhangetyy@163.com.
