# Supplementary material for: Potential causal associations between adiposity and hydration status in children and adolescents: evidence from negative control and Mendelian randomization analyses
Source: Front Nutr. 2026 Sep 28;13:1925090. doi: 10.3389/fnut.2026.1925090 (PMC13654732; doi:10.3389/fnut.2026.1925090)
Supplement: Supplementary file 1 [file Table_1.DOCX]

**Supplementary materials**

**Potential Causal Associations Between Adiposity and Hydration Status in Children and Adolescents: Evidence from Negative Control and Mendelian Randomization Analyses**

Yuwei Hou^1^, Shuying Luo^1^, Kaijuan Wang^2^, Dazhong Wang^1,*^, Yaodong Zhang^1,*^

^1^ Henan Key Laboratory of Children’s Genetics and Metabolic Diseases, Children's Hospital Affiliated to Zhengzhou University, Henan Children's Hospital, Zhengzhou Children's Hospital, Zhengzhou, Henan Province, China

^2^ Department of Epidemiology and Health Statistics, College of Public Health, Zhengzhou University, Zhengzhou, Henan Province, China

* **Correspondence to**:

Dazhong Wang, Henan Key Laboratory of Children's Genetic and Developmental Diseases, Children's Hospital Affiliated to Zhengzhou University, Henan Children's Hospital, Zhengzhou Children's Hospital, Zhengzhou 450018, Henan Province, China

E-mail addresses: 13803818341@163.com

Yaodong Zhang, Henan Key Laboratory of Children's Genetic and Developmental Diseases, Children's Hospital Affiliated to Zhengzhou University, Henan Children's Hospital, Zhengzhou Children's Hospital, Zhengzhou 450018, Henan Province, China

E-mail addresses:ydzhangetyy@163.com

**CONTENT**

**Supplementary Methods**

**Method S1**. Negative control outcome model

**Method S2**. Mendelian randomization assumptions and analytical approaches.

**Supplementary Tables**

**Table S1**. Associations between children and adolescent’s hydration status and negative outcomes.

**Table S2**. Characteristics of the SNPs used as IVs in the causal association of BMI with urine specific gravity (Supplementary Table.xlsx).

**Table S3**. Characteristics of the SNPs used as IVs in the causal association of WC with urine specific gravity (Supplementary Table.xlsx).

**Table S4**. Characteristics of the SNPs used as IVs in the causal association of urine specific gravity on BMI and WC (Supplementary Table.xlsx).

**Table S5**. Mendelian randomization estimates for the causal effects of urine specific gravity on BMI and WC

**Table S6.** Sensitivity analyses of the associations of BMI Z-scores and WC Z-scores with log-transformed USG (n = 11,935)

**Table S7.** Sensitivity analyses of the associations between BMI Z-scores, WC Z-scores and hydration status after excluding extreme values (n = 10,784)

**Table S8**. Sensitivity analyses of associations between children and adolescent’s hydration status and negative control outcomes after excluding extreme values (n = 9,744)

**Table S9**. Mendelian randomization estimates for the causal effects of urine specific gravity on BMI and WC using alternative SNP selection thresholds (SNP = 8)

**Table S10**. Characteristics of SNPs used as IVs for the causal association of urine specific gravity with BMI and WC using alternative SNP selection thresholds (Supplementary Table.xlsx).

**Supplementary Figures**

**Figure S1**. Flow diagram of participant selection.

**Figure S2**. Scatterplots of BMI and WC, and their corresponding Z-scores, in children and adolescents.

**Figure S3.** Correlation matrix among adolescent’s BMI, paternal BMI, maternal BMI, and mean parental BMI.

**Figure S4**. Subgroup analyses of the association between BMI Z-scores and hydration status in children and adolescents.

**Figure S5**. Subgroup analyses of the association between WC Z-scores and hydration status in children and adolescents.

**Figure S6**. Leave-one-out analysis for the causal effect of BMI on urine specific gravity.

**Figure S7**. Leave-one-out analysis for the causal effect of WC on urine specific gravity.

**Figure S8**. Leave-one-out analysis for the causal effect of urine specific gravity on BMI.

**Figure S9**. Leave-one-out analysis for the causal effect of urine specific gravity on WC.

**Figure S10.** Leave-one-out analysis for the causal effect of urine specific gravity on BMI using alternative SNP selection thresholds (SNP = 8)

**Figure S11.** Leave-one-out analysis for the causal effect of urine specific gravity on WC using alternative SNP selection thresholds (SNP = 8)

**Method S1. Negative control outcome model**

To formalize the negative control outcome (NCO) used in this study, we consider a simple linear structural equation model in which $A$ denotes the primary exposure (children and adolescent’s hydration status), $Y$ denotes the outcome (children and adolescent’s BMI), $W$ denotes the negative control outcomes (parental BMI), and $U$ denotes unmeasured confounders. The directed acyclic graph is shown in the figure below.

Figure. Directed acyclic graph for the negative control outcome

If the causal effects of $A$ on $Y$ and $W$ can be represented in linear form, we may write:

$$E\left( Y \mid A,U \right)=\beta_{Y0}+\beta_{YA}A+\beta_{YU}U,$$

$$E(U\mid A)=\beta_{U0}+\beta_{UA}A.$$

Substituting yields $E(Y\mid A)=\beta_{Y0}+(\beta_{YA}+\beta_{YU}\beta_{UA})A+\beta_{YU}\beta_{U0}.$

Here, $\beta_{YA}$ represents the true causal effect of $A$ on $Y$, whereas the term $\beta_{YU}\beta_{UA}$ corresponds to the bias term induced by the unmeasured confounder $U$. For the negative control outcomes $W$, we assume that $A$ has no direct causal effect on $W$; that is, $E\left( W \mid U \right)=\beta_{W0}+\beta_{WU}U,$ and $E(W\mid A,U)=E(W\mid U).$ So,

$$E\left( W | A \right)=E\left\{ E\left( W | A,U \right) | A \right\}$$

$$=E\left\{ E\left( W | U \right) | A \right\}$$

$$=E[\beta_{W0}+ \beta_{WU}U|A]$$

$$=\beta_{W0}+ \beta_{WU}E\left( U | A \right)$$

$$=\beta_{W0}+ \beta_{WU}(\beta_{U0}+\beta_{UA}A)$$

$$=\beta_{W0} + \left( \beta_{WU}\beta_{UA} \right)A + \beta_{WU} \beta_{U0},$$

shows that both associations are driven by the same unmeasured confounder through the shared term $E(U\mid A)$. Therefore, evidence of an association between $A$ and $W$ may indicate the presence of residual unmeasured confounding in the estimated association between $A$ and $Y$.

It should be noted that this diagnostic use of the negative control outcome remains valid even if parental BMI directly influences child BMI, because the derivation of $E(W\mid A)$ does not condition on $Y$. As long as the exposure $A$ has no causal effect on $W$ and $U$ can capture their shared confounding, any association between $A$ and $W$ still exclusively reflects confounding by $U$.

**Method S2.** Mendelian randomization assumptions and analytical approaches

Mendelian randomization (MR) analysis was conducted using genetic variants as instrumental variables to evaluate the potential causal relationship between adiposity-related traits and urine specific gravity (USG). A valid MR analysis relies on three core instrumental variable assumptions, as shown in the figure below.

Figure. Schematic diagram of Mendelian randomization study

First, the relevance assumption (IV1) requires that selected genetic variants are robustly associated with the exposure of interest. Second, the independence assumption (IV2) states that genetic instruments are independent of confounders of the exposure–outcome relationship. Because germline genetic variants are fixed at conception, they are theoretically independent of postnatal environmental, behavioral, and lifestyle factors, thereby reducing confounding bias. Third, the exclusion restriction assumption (IV3) requires that genetic variants influence the outcome exclusively through the exposure, without affecting the outcome via alternative biological pathways, namely horizontal pleiotropy. To evaluate potential violations of this assumption, multiple complementary MR estimators and sensitivity analyses were implemented.

The primary causal estimate was obtained using the inverse-variance weighted (IVW) method under a random-effects model, which provides the highest statistical efficiency when all instrumental variables are valid and directional pleiotropy is absent. To enhance robustness, several complementary MR approaches were additionally applied. MR-Egger regression allows for directional horizontal pleiotropy and assesses potential pleiotropic bias through the intercept term. The weighted median estimator provides consistent causal estimates when at least 50% of the instrumental weight derives from valid instruments. The contamination mixture (ConMix) method improves estimation robustness by modeling a mixture distribution of valid and invalid instruments. The MR robust adjusted profile score (MR-RAPS) approach accounts for weak instrument bias and is robust to outlier variants. The MR-pleiotropy residual sum and outlier (MR-PRESSO) method was used to detect and correct for horizontal pleiotropy by identifying and removing outlier variants. The constrained maximum likelihood and model averaging Bayesian information criterion (cML-MA-BIC) method applies a model-averaging framework to obtain reliable causal estimates in the presence of pleiotropy and invalid instruments. Consistency of results across multiple MR methods was interpreted as evidence supporting the robustness of causal inference and reducing reliance on any single modeling assumption.

**Table S1**. Associations between children and adolescent’s hydration status and negative outcomes

| Exposure | Negative outcomes | *β* | 95% *CI* | *P* |
| --- | --- | --- | --- | --- |
| Child hydration status | Paternal BMI | 0.018 | -0.101, 0.136 | 0.768 |
|  | Maternal BMI | 0.070 | -0.039, 0.178 | 0.207 |
|  | Parental mean BMI | 0.044 | -0.052, 0.140 | 0.370 |

Note: *CI*, confidence interval. All models were estimated using robust standard errors clustered at the school level to account for the multistage sampling design.

**Table S2.** Characteristics of the SNPs used as IVs in the causal association of BMI with urine specific gravity (Supplementary Table.xlsx).

**Table S3.** Characteristics of the SNPs used as IVs in the causal association of WC with urine specific gravity (Supplementary Table.xlsx).

**Table S4.** Characteristics of the SNPs used as IVs in the causal association of urine specific gravity on BMI and WC (Supplementary Table.xlsx).

**Table S5**. Mendelian randomization estimates for the causal effects of urine specific gravity on BMI and WC

| Outcomes | Methods | *β* (95% *CI*) | *P* | *Q* | *I^2^* | Intercept |
| --- | --- | --- | --- | --- | --- | --- |
| BMI | IVW | -0.038 (-0.101, 0.026) | 0.245 | 39.474 | 64.53% | -0.0001  (*P*= 0.986) |
|  | MR-Egger | -0.037 (-0.159, 0.085) | 0.564 |  |  |  |
|  | Weighted median | -0.040 (-0.097, 0.016) | 0.170 |  |  |  |
|  | ConMix | -0.059 (-0.109, 0.001) | 0.087 |  |  |  |
|  | MR-PRESSO | -0.008 (-0.061, 0.045) | 0.769 |  |  |  |
|  | MR-RAPS | -0.034 (-0.088, 0.020) | 0.211 |  |  |  |
|  | cML-MA-BIC | -0.015 (-0.072, 0.041) | 0.591 |  |  |  |
| WC | IVW | -0.004 (-0.057, 0.049) | 0.873 | 29.082 | 51.86% | 0.001  (*P*= 0.627) |
|  | MR-Egger | -0.026 (-0.126, 0.075) | 0.623 |  |  |  |
|  | Weighted median | 0.002 (-0.053, 0.057) | 0.945 |  |  |  |
|  | ConMix | 0.026 (-0.064, 0.076) | 0.394 |  |  |  |
|  | MR-PRESSO | 0.004 (-0.037, 0.045) | 0.851 |  |  |  |
|  | MR-RAPS | -0.004 (-0.054, 0.045) | 0.863 |  |  |  |
|  | cML-MA-BIC | 0.003 (-0.044, 0.051) | 0.890 |  |  |  |

Note: *CI*, confidence interval; cML-MA-BIC, constrained maximum likelihood and model averaging Bayesian information criterion; ConMix, contamination mixture; IVW, inverse-variance weighted; MR-PRESSO, MR pleiotropy residual sum and outlier; MR-RAPS, MR robust adjusted profile score.

**Table S6**. Sensitivity analyses of the associations of BMI Z-scores and WC Z-scores with log-transformed USG (n = 11,935)

| Model | BMI Z-scores | | | WC Z-scores | | |
| --- | --- | --- | --- | --- | --- | --- |
|  | *β* | 95% *CI* | *P* | *β* | 95% *CI* | *P* |
| Model 1 | 0.0004 | -0.0002, 0.0008 | 0.063 | 0.0005 | 0.0002, 0.0007 | 0.006 |
| Model 2 | 0.0013 | 0.0004, 0.0022 | 0.005 | 0.0004 | 0.0002, 0.0006 | <0.001 |
| Model 3 | 0.0013 | 0.0004, 0.0022 | 0.005 | 0.0004 | 0.0002, 0.0006 | <0.001 |
| Model 4 | 0.0006 | 0.0002, 0.0010 | 0.003 | 0.0005 | 0.0002, 0.0007 | <0.001 |

Note: *CI*, confidence interval. Model 1 was unadjusted. Model 2 was fitted with adjustment for covariates including sex, age, parental education, income, breakfast frequency, screen time, sleep duration, weekly exercise frequency, and total exercise duration. Model 3 additionally adjusted for late-night eating habits and snack consumption habits beyond Model 2 covariates. Model 4 was based on a stabilized IPW model incorporating all covariates. All models were estimated using robust standard errors clustered at the school level to account for the multistage sampling design.

**Table S7**. Sensitivity analyses of the associations between BMI Z-scores, WC Z-scores and hydration status after excluding extreme values (n = 10,784)

| Model | BMI Z-scores | | | WC Z-scores | | |
| --- | --- | --- | --- | --- | --- | --- |
|  | *OR* | 95% *CI* | *P* | *OR* | 95% *CI* | *P* |
| Model 1 | 1.152 | 0.921 - 1.440 | 0.215 | 1.232 | 1.096 - 1.385 | <0.001 |
| Model 2 | 1.537 | 0.984 - 2.401 | 0.059 | 1.226 | 1.104 - 1.362 | <0.001 |
| Model 3 | 1.521 | 0.975 - 2.372 | 0.064 | 1.223 | 1.102 - 1.357 | <0.001 |
| Model 4 | 1.663 | 1.078 - 2.564 | 0.022 | 1.235 | 1.111 - 1.373 | <0.001 |

Note: *OR*, odds ratio; *CI*, confidence interval. Model 1 was unadjusted. Model 2 was fitted with adjustment for covariates including sex, age, parental education, income, breakfast frequency, screen time, sleep duration, weekly exercise frequency, and total exercise duration. Model 3 additionally adjusted for late-night eating habits and snack consumption habits beyond Model 2 covariates. Model 4 was based on a stabilized IPW model incorporating all covariates. All models were estimated using robust standard errors clustered at the school level to account for the multistage sampling design.

**Table S8**. Sensitivity analyses of associations between children and adolescent’s hydration status and negative control outcomes after excluding extreme values (n = 9,744)

| Exposure | Negative outcomes | *β* | 95% *CI* | *P* |
| --- | --- | --- | --- | --- |
| Child hydration status | Paternal BMI | -0.045 | -0.171, 0.082 | 0.488 |
|  | Maternal BMI | 0.045 | -0.100, 0.190 | 0.540 |
|  | Parental mean BMI | 0.001 | -0.114, 0.114 | 0.996 |

Note: *CI*, confidence interval. All models were estimated using robust standard errors clustered at the school level to account for the multistage sampling design.

**Table S9**. Mendelian randomization estimates for the causal effects of urine specific gravity on BMI and WC using alternative SNP selection thresholds (SNP = 8)

| Outcomes | Methods | *β* (95% *CI*) | *P* | *Q* | *I^2^* | Intercept |
| --- | --- | --- | --- | --- | --- | --- |
| BMI | IVW | -0.019 (-0.09, 0.053) | 0.607 | 13.842 | 49.43% | 0.004  (*P*= 0.279) |
|  | MR-Egger | -0.084 (-0.212, 0.044) | 0.246 |  |  |  |
|  | Weighted median | -0.041 (-0.111, 0.029) | 0.254 |  |  |  |
|  | ConMix | -0.079 (-0.159, 0.131) | 0.149 |  |  |  |
|  | MR-PRESSO | -0.018 (-0.090, 0.053) | 0.623 |  |  |  |
|  | MR-RAPS | -0.034 (-0.103, 0.034) | 0.324 |  |  |  |
|  | cML-MA-BIC | -0.020 (-0.082, 0.042) | 0.529 |  |  |  |
| WC | IVW | -0.008 (-0.081, 0.065) | 0.830 | 15.436 | 54.65% | 0.001  (*P*= 0.734) |
|  | MR-Egger | -0.030 (-0.173, 0.113) | 0.698 |  |  |  |
|  | Weighted median | 0.010 (-0.059, 0.079) | 0.776 |  |  |  |
|  | ConMix | 0.036 (-0.054, 0.116) | 0.166 |  |  |  |
|  | MR-PRESSO | 0.020 (-0.036, 0.075) | 0.517 |  |  |  |
|  | MR-RAPS | -0.010 (-0.078, 0.058) | 0.767 |  |  |  |
|  | cML-MA-BIC | 0.001 (-0.063, 0.066) | 0.964 |  |  |  |

Note: BMI, body mass index; *CI*, confidence interval; cML-MA-BIC, constrained maximum likelihood and model averaging Bayesian information criterion; ConMix, contamination mixture; IVW, inverse-variance weighted; MR-PRESSO, MR pleiotropy residual sum and outlier; MR-RAPS, MR robust adjusted profile score; WC, waist circumference.

**Table S10.** Characteristics of SNPs used as IVs for the causal association of urine specific gravity with BMI and WC using alternative SNP selection thresholds (Supplementary Table.xlsx).

**Figure S1**. Flow diagram of participant selection.

**Figure S2**. Scatterplots of BMI and WC, and their corresponding Z-scores, in children and adolescents.

**Figure S3**. Correlation matrix among adolescent’s BMI, paternal BMI, maternal BMI, and mean parental BMI.

**Figure S4**. Subgroup analyses of the association between BMI Z-scores and hydration status in children and adolescents.

**Figure S5**. Subgroup analyses of the association between WC Z-scores and hydration status in children and adolescents.

**Figure S6.** Leave-one-out analysis for the causal effect of BMI on urine specific gravity

**Figure S7.** Leave-one-out analysis for the causal effect of WC on urine specific gravity

**Figure S8.** Leave-one-out analysis for the causal effect of urine specific gravity on BMI

**Figure S9.** Leave-one-out analysis for the causal effect of urine specific gravity on WC

**Figure S10.** Leave-one-out analysis for the causal effect of urine specific gravity on BMI using alternative SNP selection thresholds (SNP = 8)

**Figure S11.** Leave-one-out analysis for the causal effect of urine specific gravity on WC using alternative SNP selection thresholds (SNP = 8)
